# Late Diagnosis of Pregnancy in a Transmasculine Patient on Testosterone: A Case Report and Patient Perspective

**DOI:** 10.7759/cureus.91315

**Published:** 2025-08-30

**Authors:** Nupur Agrawal, Maralee Kanin, Hazel L Binder, Alexandra Havard, Shira Grock

**Affiliations:** 1 Department of Medicine, Division of Internal Medicine and Pediatrics, University of California Los Angeles David Geffen School of Medicine, Los Angeles, USA; 2 Department of Medicine, Division of Endocrinology, Diabetes and Metabolism, University of California Los Angeles David Geffen School of Medicine, Los Angeles, USA; 3 Associate Degree Program, Santa Monica College, Santa Monica, USA; 4 Department of Obstetrics and Gynecology, Kaiser Permanente, Bakersfield, USA

**Keywords:** estradiol, gender-affirming care, methods of contraception, pregnancy detection, testosterone

## Abstract

Testosterone therapy can be an important component of care for transgender and gender-diverse (TGD) patients assigned female at birth (AFAB) who wish to pursue hormonal transition. While testosterone often leads to menstrual suppression, it is not a reliable source of contraception and can be teratogenic. Our patient presented to the clinic at age 18 to establish care and resume gender-affirming hormone therapy with testosterone (GAHT-T). The patient's first visit, typically a comprehensive intake conducted to understand patients' general health and transition-related goals, which includes discussion surrounding sexual health and family planning, was truncated due to time constraints. However, a joint decision was made to restart GAHT-T given its importance for the patient's health and transition. Routine labs to assess hormone levels were conducted at follow-up and incidentally showed rising estradiol levels, which are unusual for patients on GAHT-T, and triggered concern and evaluation for a possible underlying endocrine tumor. The patient was ultimately determined to have a single intrauterine pregnancy at 35 weeks of gestation. Understanding patients’ comprehensive health and transition-related goals and routinely engaging in shared decision-making can optimize options, autonomy, well-being, and health outcomes for TGD patients. Providers should be skilled in sensitively and meaningfully engaging patients in conversations surrounding overall health, sexual health, contraception counseling, family planning, fertility preservation, and options when there is a late diagnosis of pregnancy. Our patient shares insight on the physical and psychological impact of their journey and the significance of open and safe patient-provider relationships in supporting whole health for patients.

## Introduction

Testosterone therapy can be an important component of care for transgender and gender-diverse (TGD) patients assigned female at birth (AFAB) who wish to pursue hormonal transition. Testosterone is a known teratogen that can cause virilization of a fetus with XX chromosomes [1] and does not serve as adequate contraception. Thus, counseling on sexual health and contraception is critical. Guidelines provide no consensus on the extent of counseling that should be provided and make no mention of whether providers should consider baseline pregnancy tests for individuals having sex in a way that can result in pregnancy [2,3]. This allows for variable practice among healthcare providers and may perpetuate existing barriers to accessing appropriate and comprehensive reproductive and sexual healthcare.

## Case presentation

Our patient presented to the clinic at age 18 to establish care and reinitiate gender-affirming hormone therapy with testosterone (GAHT-T) after experiencing a lapse in care. The patient was AFAB, identified as a transgender male, and used they/them pronouns. They knew they were transgender their whole life but did not have the terminology to express their gender to others until ninth grade. They previously completed leuprolide therapy and were on GAHT-T, which was interrupted several months prior due to insurance changes. Upon presentation to our clinic, they were eager to restart GAHT-T. Relevant past medical history included a body mass index (BMI) of 44.14 kg/m^2^ and anxiety and depression that were well managed with therapy.

**Figure 1:**
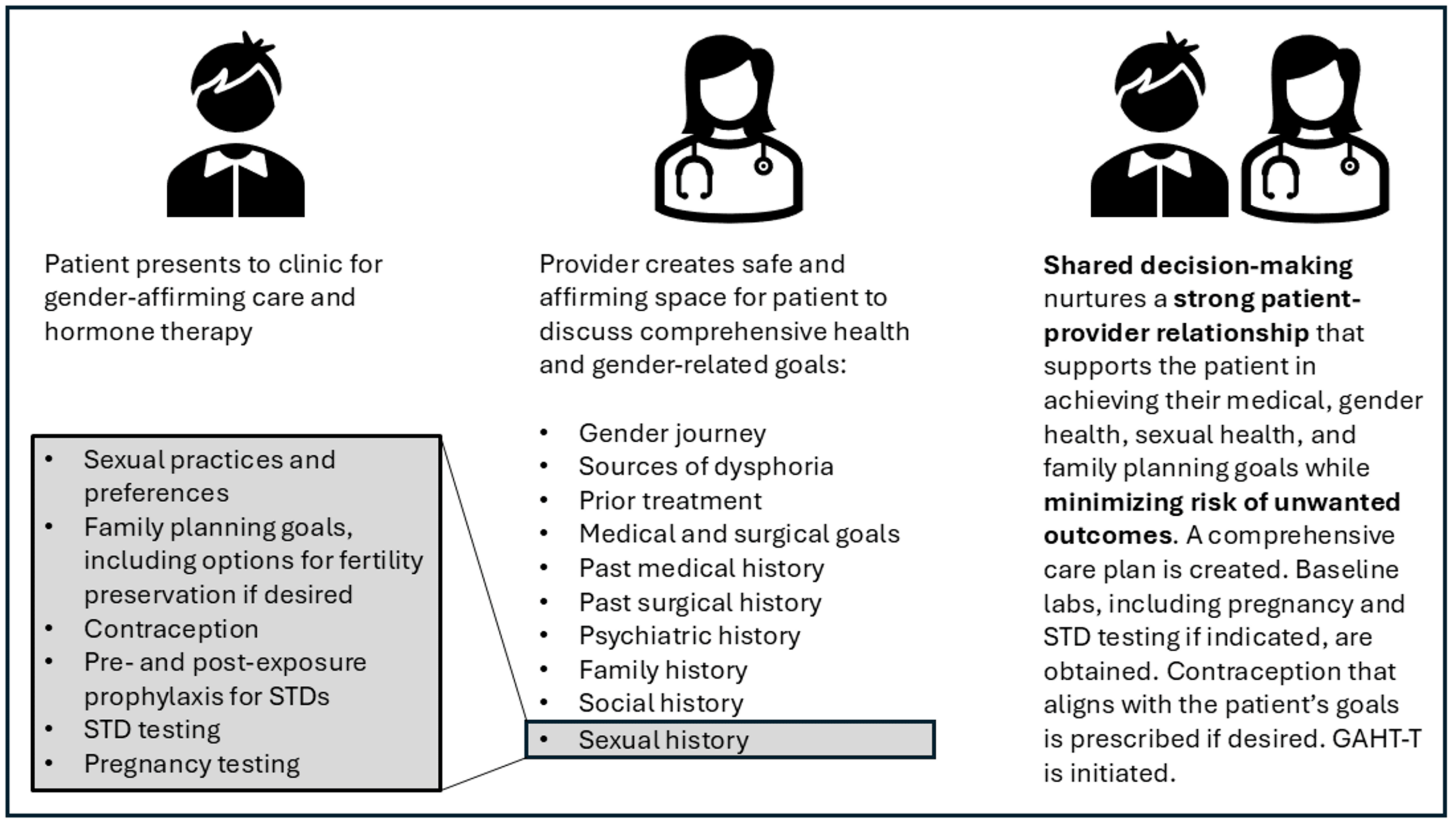
Engaging in shared decision-making with AFAB patients interested in GAHT-T AFAB: assigned female at birth, GAHT-T: gender-affirming hormone therapy with testosterone. This figure was created by the authors and is original work developed specifically for this case study.

A detailed intake for patients initially presenting to our clinic is offered and encouraged (Figure 1), so we can fully understand patients’ general health and transition-related goals. Our patient opted to defer this comprehensive intake due to time constraints but expressed their need to restart GAHT-T. The patient had previously been on GAHT-T without issues and was experiencing dysphoria after having to abruptly stop hormone therapy. They expressed that their transition was the most important aspect of their overall health and that restarting GAHT-T was essential for their well-being. They denied any new health concerns since last being on hormone therapy. A joint decision was made to restart GAHT-T at that visit with a plan to conduct labs and discuss the patient's health history and health-related goals in greater detail at subsequent visits.

The patient followed up eight weeks after restarting GAHT-T at which time hormone analysis revealed testosterone levels on the low end for GAHT-T and markedly elevated estradiol levels (Table 1). Repeat levels four weeks later showed rising estradiol levels, which prompted urgent referral to endocrinology.

Upon presentation to the endocrinology clinic, the patient denied vaginal bleeding and chest (breast) tenderness. The date of their last menstrual period was reported as two years prior. Physical exam was notable for BMI 42.6 kg/m², mild facial hair development, and central adiposity. Comprehensive endocrinology labs were performed, and estradiol levels were confirmed to be elevated by mass spectrometry (Table 1). The limited differential diagnosis for elevated estradiol levels in AFAB individuals on GAHT-T includes rare estrogen-secreting tumors, pregnancy, and lab error [4,5]. Given the concern for possible malignancy, a computed tomography (CT) scan of the abdomen/pelvis was ordered. Prior to completing the CT, additional labs revealed elevated alpha fetoprotein, progesterone, inhibin A, and beta-hCG (Table 1). The beta-hCG prompted a more thorough sexual health history. The patient confirmed having sex in a way that could result in pregnancy. The CT was cancelled, and an abdominal ultrasound was obtained, which revealed a single intrauterine pregnancy with an estimated gestational age of 35 weeks 2 days.

**Table 1:** Laboratory test results GAHT-T: gender-affirming hormone therapy with testosterone, LH: luteinizing hormone, FSH: follicle-stimulating hormone, AMH: anti-Müllerian hormone, CA-125: cancer antigen 125, DHEA: dehydroepiandrosterone, hCG: human chorionic gonadotropin.

Laboratory Test	Result	Reference Range
Initial labs drawn eight weeks after reinitiation of GAHT-T		
Testosterone	172	200-1000 ng/dL cis-male
Estradiol	11,160	80-400 pg/mL mid-cycle
Follow-up labs drawn 4 weeks later		
Estradiol	17,486	80-400 pg/mL mid-cycle
Initial endocrinology labs		
Estradiol	21,265	80-400 pg/mL mid-cycle
Estradiol by mass spectrometry	>10,000	100-400 pg/mL late follicular
Prolactin	328	2.8-29.2 cis-female
LH	<0.1	10-91 mIU/mL mid-cycle
FSH	<0.1	6-23 mIU/mL mid-cycle
Anti-mullerian hormone	0.568	0.401-16.015 ng/mL cis-female 18-29 years
Androstenedione	1.825	0.26-2.14 ng/mL cis-female 18-39 years
CA-125	19	<38 U/mL
DHEA-sulfate	2,460	400-3600 ng/ml cis-female
Follow-up endocrinology labs		
Alpha-fetoprotein	126	0-6.7 ng/mL
Progesterone	100.2	3.0-25.0 ng/mL luteal phase
Inhibin A	500.1	16.9-91.8 pg/mL
Beta-hCG	32,571	<4 mIU/mL non-pregnant cis-female

Once pregnancy was confirmed, the patient was counseled regarding the risks of testosterone exposure to the fetus and the implications of late entry to prenatal care. Testosterone was immediately discontinued, and the patient was urgently scheduled with obstetrics, maternal-fetal medicine, and psychology. Detailed fetal anatomic ultrasound revealed a viable fetus with XX chromosomes and no obvious anatomic defects or evidence of virilization. Table 2 details the interdisciplinary healthcare team ultimately involved in the patient's care.

**Table 2:** Healthcare team members and their roles in caring for an AFAB patient on GAHT-T with late diagnosis of pregnancy GAHT-T: gender-affirming hormone therapy with testosterone, AFAB: assigned female at birth.

Provider	Role
Primary care provider	Conducted initial intake and follow up visits with patient
	Engaged in shared-decision making with patient to develop comprehensive care plan, including general and gender health-related goals
	Prescribed and managed GAHT-T
	Ordered and interpreted GAHT-T monitoring labs
	Referred patient to endocrinology after serial labs showed elevated estradiol levels
	Coordinated care for patient with specialists once pregnancy was confirmed
	Provided ongoing support to patient after delivery
Endocrinologist	Conducted endocrinology intake with patient
	Ordered lab and imaging studies to determine underlying cause of elevated estradiol levels
	Performed follow up history to investigate cause of elevated beta-hCG levels and diagnosed pregnancy
General obstetrician and gynecologist	Conducted prenatal and postnatal visits with patient
	Coordinated delivery
Maternal-fetal medicine specialist	Performed fetal anatomy scan
Psychologist	Conducted prenatal psychosocial assessment and provided targeted support to patient
Administrative staff	Supported patient in scheduling primary care, subspecialty, and therapy visits after diagnosis of pregnancy

After the diagnosis of pregnancy, the patient faced limited options for perinatal decisions and limited time to prepare for postpartum considerations. However, they went on to have an uncomplicated normal spontaneous vaginal delivery of a viable AFAB infant at 37 weeks 2 days with no apparent abnormalities or virilization. The patient was seen by social work and lactation postpartum and chose to remain off GAHT-T to chest feed the baby. The patient's partner and family have been very supportive in caring for the baby. Their child is now six years old and thriving.

## Discussion

Our patient's case highlights the need for robust counseling, guidance, and access to sexual and reproductive healthcare for AFAB individuals on GAHT-T. Significant barriers exist that limit providers’ ability to provide sufficient counseling [6], but there are still multiple opportunities to address family planning and contraceptive options prior to and during GAHT-T use.

Conducting a detailed intake and baseline labs prior to starting GAHT is advantageous because it allows the patient and provider to establish a care plan that supports the patient’s health in a holistic manner. In our patient’s case, it would have helped the primary care provider learn that the patient was sexually active and at risk for pregnancy, triggering a conversation on safe sex practices, family planning, contraception, and possibly pregnancy testing. Thorough knowledge of hormonal and non-hormonal contraception, access to routine pregnancy and STD testing, and encouragement to engage in these conversations can empower patients to make well-informed decisions on family planning.

Patients value our team’s comprehensive approach to patient care and the opportunity to discuss in detail the risks, benefits, and alternative treatments available to them as part of their transition. The most notable disadvantage of performing a detailed intake prior to prescribing GAHT is that it may be perceived as gatekeeping, which can be problematic as TGD patients already face numerous barriers to care, including delays in appropriate healthcare and suboptimal health outcomes. We address this by engaging in informed decision-making with patients and encouraging them to take a proactive role in their care.

Even when providers have the opportunity to conduct a detailed intake, many lack the knowledge and comfort needed to engage TGD patients in informed discussions about reproductive health. They may be unsure of gender-inclusive terminology for body parts, processes, and procedures and may alienate patients by using inappropriate terminology [6]. Providers may lack the time needed to facilitate a thorough conversation on contraception options. Specifically, they may find it difficult to address questions regarding the impact of estrogen-containing contraceptive options on transition, which is often of paramount concern to patients. Limited research suggests that AFAB individuals perceive their providers as lacking knowledge regarding pregnancy prevention [7]. Patients may fear discrimination, misgendering, and a lack of confidentiality [8]. These barriers lead to missed opportunities to provide counseling and pregnancy testing when appropriate and to address patient goals and questions, which can result in inconsistent or inadequate contraceptive practices.

Providers should reassure patients that appointments are confidential and protected by the Health Insurance Portability and Accountability Act (HIPAA). Extra care should be taken to ensure that patients’ gender history is not shared with anyone outside the medical team and that the medical record appropriately reflects their lived name, gender identity, and pronouns.

Counseling about contraception options should be individualized based on patient preference and risk factors. Providers should have an understanding of patients’ identity and their social situation to engage in shared decision-making [6]. In those having sex in a way that could result in pregnancy, preventive counseling is imperative given the lower frequency of contraceptive use in the TGD population [7]. Testosterone often leads to menstrual suppression, but patients and providers may not understand that, even with amenorrhea, testosterone is not a reliable source of contraception [9]. This, along with the inconsistent use of contraception, puts patients at higher risk for undesired pregnancy. There should also be a low threshold to provide pregnancy testing when indicated. Beta-hCG testing obtained via blood may be less dysphoria-inducing than a urine pregnancy test.

Shared decision-making should also be used to address contraceptive options, including benefits, limitations, and how their use may affect transition goals. The major classes of hormonal and non-hormonal birth control are explored in Table 3 [10]. To our knowledge, extensive data on contraception usage, safety, and efficacy among this population are lacking. There is limited data on pharmacologic interactions between hormonal contraception and GAHT-T. It is unclear whether estrogen-containing birth control counteracts the effects of testosterone, and use of these agents could worsen dysphoria for some. Progesterone-only contraceptives may be preferred by patients who do not want to administer estrogen, but these have limitations in terms of invasiveness of placement (e.g., intrauterine devices require a gynecologic exam for placement) and regularity of use for desired contraceptive effects. The process of placing other forms of birth control, such as vaginal rings, intrauterine devices, or diaphragms, may also worsen dysphoria. Ultimately, contraceptive options should be discussed in the context of patients’ individual transition and family planning goals.

**Table 3:** Contraceptive options and considerations for AFAB TGD patients DMPA: depot medroxyprogesterone acetate, IM: intramuscular, SQ: subcutaneous, IUS: intrauterine system, AFAB: assigned female at birth, TGD: transgender and gender diverse.

Medication	Dosing	Effectiveness (in Cisgender Women)	Potential Benefits	Potential Risks/Concerns in TGD Individuals
Progesterone-only options				
Depot medroxyprogesterone acetate (DMPA, Depo Provera, Depo-Subq)	150 mg IM every 11-15 weeks or 104mg SQ every 11-15 weeks	94%	High rates of amenorrhea, no estrogen, self-administration possible	Weight gain, delayed return of menses/fertility upon discontinuation, decreased bone mineral density
Norethindrone (Micronor, Errin, and others)	0.35 mg oral once daily	91%	Once daily oral administration, inexpensive and readily covered by insurance	Higher rates of breakthrough bleeding, daily dosing, less effective compared to other contraceptive methods
Levonorgestrel intrauterine system (IUS) (Mirena, Skyla, Kyleena, Liletta)	Varied dosing; provides continuous release of levonorgestrel for up to 8 years	99.8%	Long-acting and reversible, with minimal user interaction once placed, effective menstrual suppression	Potentially painful and dysphoric insertion process that requires pelvic and genital exam; procedure-related pain, bleeding, uterine perforation, infection
Etonogestrel subdermal implant (Nexplanon)	68 mg device placed subdermally every 3 years	99.95%	Long-acting, reversible contraception	Higher rates of breakthrough bleeding; procedure-related pain, bleeding, and infection
Estrogen-containing options				
Combined hormonal contraception pill (many formulations and brands)	Varies but typically 1 pill orally once daily	91%	Once daily oral administration, widely available, inexpensive	Contains estrogen, which could trigger dysphoria, increased thrombogenic risk, and daily dosing
Combined hormonal contraceptive patch (many formulations and brands)	Ethinyl estradiol and norelgestromin system placed transdermally once weekly for 3 consecutive weeks, followed by a patch-free week, then repeated	91%	Longer-acting, widely available, easy administration	Contains estrogen, which could trigger dysphoria, increased thrombogenic risk, dermatitis, and may be visible to others depending on the location of placement
Combined hormonal contraception vaginal ring (NuvaRing)	Ethinyl estradiol and etonogestrel vaginal ring is placed into the vagina for 21 days, removed for 7 days, then repeated	91%	Longer-acting, widely available, and least frequent administration of all combined contraceptive options	Requires vaginal placement and contains estrogen, which could trigger dysphoria, increased thrombogenic risk
Non-hormonal options (these options do not provide menstrual suppression)				
Copper intrauterine device system (Paragard)	1 device placed intrauterine for up to 10 years	99%	Long-acting and reversible, hormone-free, can be used as emergency contraception within 5 days of unprotected intercourse	Does not suppress menstruation; procedure-related dysphoria, pain, bleeding, uterine perforation, infection

Unplanned pregnancy presents diagnostic and management challenges. Amenorrhea is often the first clue to detecting pregnancy in a clinical setting; however, the majority of TGD patients on testosterone experience cessation of menses, thus limiting the use of amenorrhea as a diagnostic tool [10]. Providers may also have implicit bias against considering pregnancy in TGD patients, influenced by incorrect presumptions around sexual activity status, appearance and gender expression, sexual preference, and other related factors. If a pregnancy is diagnosed in an individual on testosterone, immediate referral to high-risk obstetrics should be made for perinatal care and to assess for fetal virilization. Patients with pregnancies diagnosed later in gestation may lack sufficient perinatal care and experience fewer options for early termination, delivery, and chestfeeding preparation if desired.

Current standards of care for TGD individuals on GAHT-T provide minimal guidance on contraception and conception counseling. Our patient's experience highlights the need for understanding potential physician bias, shared decision-making with patients on pregnancy testing, appropriate knowledge of contraception options, and the physical and psychological risks of unintended pregnancy. Strong patient-physician relationships entrenched in trust, compassion, and open pathways of communication can support patients in achieving their sexual health and family planning goals while minimizing the risk of unplanned pregnancies.

Patient perspective

My GAHT-T had a six-week lapse when I switched from a pediatric to an adult provider; I became pregnant in that window without knowing it. When I went to my new physician’s office to establish care and restart testosterone, I was not able to fully complete the process of getting to know my doctor and obtaining the recommended blood work due to logistical issues.

After restarting testosterone, I returned to see my doctor to monitor my hormone levels. The initial labs showed high estrogen levels that needed to be rechecked. I assumed the lab had made a mistake but felt worse when I found out my repeat estrogen levels were even higher than before. More tests showed estrogen levels in the thousands, and I was told these were concerning for a tumor. I was terrified that I was going to need surgery. In discussing next steps, we realized I was pregnant. The doctors on my care team referred me to OBGYN, where we confirmed that my baby was healthy and without abnormalities. After OB appointments almost every other day for about one month, I delivered my daughter, who was perfectly healthy and happy.

I had gone almost the full length of my pregnancy not knowing I was pregnant and felt like a horrible parent as a result. It was difficult explaining to others that I did not know I was pregnant: I had no idea what it felt like to be pregnant and had easily attributed the fatigue to viral colds, the headaches to lack of sleep, and the weight changes to a desire to be healthier but not always being able to access a nutritious diet as a high school student. The thought of pregnancy never crossed my mind.

Aside from a quick overview of sex in the sixth grade, I never received a safe sex talk and could not discuss the topic with my religious family, who believe in an abstinence-only approach until marriage. Having had to rush to prepare myself mentally and physically to be a parent and care for another human being, I believe the patient-physician relationship is a crucial space to discuss sexual health. Sexual activity should be assumed, and pregnancy should always be a consideration in patients able to become pregnant regardless of transition status. Physicians should let patients know that they have the patients’ best interests at heart and are in their corner. These discussions should occur in private without parents present, and physicians should make patients feel heard and in control of their decisions. Appropriate care steps (e.g., possible need for a pregnancy test) should be explained in understandable terms, and options should be provided when possible. Patients come from different family backgrounds and support systems, so creating a safe space where they can be open and honest will support their care long-term. These steps can prevent others from going through the fear and trauma I experienced and give them more options for their care.

## Conclusions

Understanding patients’ comprehensive health and transition-related goals and routinely engaging in shared decision-making can optimize options, autonomy, well-being, and health outcomes for TGD patients. Providers should be skilled in sensitively and meaningfully engaging patients in conversations surrounding overall health, sexual health, contraception counseling, and options available when there is a late diagnosis of pregnancy.
